# Linagliptin: A thorough Characterization beyond Its Clinical Efficacy

**DOI:** 10.3389/fendo.2013.00016

**Published:** 2013-02-26

**Authors:** Maria Angela Sortino, Tiziana Sinagra, Pier Luigi Canonico

**Affiliations:** ^1^Department of Clinical and Molecular Biomedicine, Section of Pharmacology and Biochemistry, University of CataniaCatania, Italy; ^2^Department of Scienze del Farmaco, University of Piemonte OrientaleNovara, Italy

**Keywords:** diabetes mellitus, incretin, DPP-4 inhibitors, anti-diabetic drugs, GLP-1

## Abstract

Linagliptin, one of the five dipeptidyl peptidase-4 inhibitors available, has recently entered the market both in the US and in most European countries for treatment of type 2 diabetes mellitus. It presents a xanthine-based structure, and is characterized by unique pharmacokinetics, with non-linear profile, long terminal half-life allowing prolonged exposure to the drug. It is eliminated predominately through the intestinal tract and only minimally into urine, so that it can be administered, without any dose adjustment, in conditions of renal impairment. Linagliptin is effective in modifying all parameters of hyperglycemia either in monotherapy, or as add-on therapy, together with metformin or a sulfonylurea. It also exhibits a good tolerability profile with few side effects, absence (when used in monotherapy), or low risk (when in combination with a sulfonylurea) of hypoglycemia. More importantly it has a weight neutral effect. A comprehensive report of the literature on linagliptin is provided, paying attention in particular to preclinical studies, interactions with other drugs, safety and tolerability, and results obtained in animal models that highlight properties of linagliptin suggestive of potential additional uses. Particularly promising appear the data demonstrating a positive effect of linagliptin on metabolic dysfunction and renal and/or cardiovascular damage together with more recently reported effects of linagliptin on tissue repair and neuroprotection.

## Introduction

The devastating prevalence of type 2 diabetes mellitus (T2DM), together with better knowledge of pathogenesis of the disease, have prompted search for new treatment strategies. Dipeptidyl peptidase-4 (DPP-4) inhibitors offer novel opportunities for the management of T2DM as they are endowed with a new mechanism of action, potentiating the physiological function of endogenous incretin, glucagon-like peptide 1 (GLP-1). Although cleavage of GLP-1 is recognized as the only clinically relevant enzymatic effect of DPP-4, this enzyme recognizes as target glucose-dependent insulinotropic polypeptide (GIP), another incretin that shares insulinotropic activity with GLP-1, but is devoid of other properties such as reduction of glucagon release, delay of gastric emptying, and suppression of appetite (Baggio and Drucker, 2007). In addition, besides incretins, other substrates of DPP-4, not directly involved in the control of glycemic homeostasis have been identified. These include different peptides with recognized renal and cardiovascular effects such as Brain Natriuretic Peptide (BNP), Neuropeptide Y (NPY), Peptide YY (PYY), and type 1 Stromal-Derived Factor (SDF-1α; Hocher et al., 2012a). Although we are far from identifying a direct link between inhibition of DPP-4, modified cleavage of these peptides and clinical correlates, it cannot be excluded that changes in their metabolism account for additional outcomes of DPP-4 inhibition that are increasingly emerging from preclinical studies.

Drugs belonging to the class of DPP-4 inhibitors are virtually devoid of any hypoglycemic effect and have no significant impact on body weight. In addition, with respect to other drugs belonging to the incretin family, DPP-4 inhibitors offer the undeniable advantage of oral administration. Five DDP-4 inhibitors are available, including sitagliptin, saxagliptin, vildagliptin, and the more recent linagliptin and alogliptin (Russell-Jones and Gough, 2012).

At present sitagliptin, linagliptin and saxagliptin are marketed in US, sitagliptin, saxagliptin, and vildagliptin have been approved in Europe and alogliptinis already present only in the Japanese market and has just received approval by US Food and Drug Administration (FDA). Linagliptin represents the most recently approved of this class of anti-diabetic drugs both in US and Europe.

Rather than analyzing the clinical properties of linagliptin, when used either as monotherapy or as add-on therapy to other anti-diabetic drugs, this review is aimed at emphasizing specific features of this molecule from preclinical studies to pharmacokinetics and interaction with other drugs. Particular attention is given to linagliptin effect in animal models, as this may represent a unique opportunity to know in depth features of the molecule that can help to disclose properties not so evident from clinical studies.

## Chemistry

(8-(3-(R)-Aminopiperidin-1-yl)-7-but-2-ynyl-3-methyl-1-(4-methyl-quinazolin-2-ylmethyl)-3,7dihydropurine-2,6-dione; linagliptin) is a DPP-4 inhibitor with a xanthine-based structure. Its nature makes it different from other compounds of the same class (saxagliptin, sitagliptin, vildagliptin, with the exception of alogliptin) that are all peptidomimetic molecules.

Linagliptin binds tightly to the core of the DPP-4 enzyme forming three hydrogen bonds between the amino function on the piperidine ring and acceptor groups on residues Glu205, Glu206, Tyr662. A fourth hydrogen bond is formed between the C-6 carbonyl of the xanthine scaffold and the backbone amide of residue Tyr631. Finally, aromatic stacking interactions are established between the xanthine ring and Tyr547 as well as between the quinazoline ring and Trp629 (Eckhardt et al., 2007).

## Pharmacology: Pharmacodynamics

Linagliptin inhibits DPP-4 activity *in vitro* with an IC50 value of 1 nM, exhibiting a potency higher than all other DDP-4 inhibitors which yield IC50 values of 19 (sitagliptin), 24 (alogliptin), 50 (saxagliptin), and 62 (vildagliptin) nM, respectively (Thomas et al., 2008). Maximal efficacy for DPP-4 inhibition is similar for linagliptin and other compounds of the same class. The kinetics of linagliptin interaction with DPP-4 reveals a slow dissociation from the enzyme with a calculated *k*_off_ rate (3 × 10^−5^/s) that is approximately 10-fold slower than the off-rate for vildagliptin (Thomas et al., 2008).

Linagliptin exhibits a 10,000-fold higher selectivity for DPP-4 than for other dipeptidyl peptidases such as DPP-2, DPP-8, and DPP-9, a property that makes this molecule, together with alogliptin, the highest selective compound vs. DPP-4. Similarly, the selectivity of linagliptin vs. peptidases N and P, prolyl oligopeptidases, and proteases such as trypsin, plasmin, and thrombin is much more than 10,000-fold. Linagliptin also inhibits fibroblast activating protein (FAP) with an IC50 of 89 nM (about 90-fold higher selectivity vs. DPP-4; Gao et al., 2007; Thomas et al., 2008; Deacon, 2011). This effect appears interesting as FAP is a protease with high potentials: it is considered in fact a marker of fibroblasts in tumor growth and is also expressed in activated fibroblasts involved in tissue remodeling (O’Brien and O’Connor, 2008). Whether and how the inhibition of FAP may contribute to the clinical effects of linagliptin is not known at present, but it is of interest that linagliptin exerts a role in reparative processes following wound injury.

## Pre-Clinical Studies

### Pharmacokinetics

Pharmacokinetics of linagliptin is characterized by its binding to the DPP-4 target, not only in plasma, but mainly in tissues, where the enzyme is bound to membranes (Greischel et al., 2010). After single or repeated oral administration in male Fischer rats, linagliptin is highly present in kidney, liver, and lung, coincident with the distribution of DPP-4 (Fuchs et al., 2009b; Retlich et al., 2009). Absorption of linagliptin is modified by intestinal P-glycoprotein. In Wistar rats receiving a single oral dose of linagliptin, administration of a P-glycoprotein inhibitor produced an increased bioavailability of the drug (Fuchs et al., 2009a), suggesting that P-glycoprotein activity limits absorption of linagliptin.

Intravenous administration produces a non-linear increase in linaglipin tissue concentrations with increasing doses, suggesting the presence of saturable binding sites. These correspond with peripheral DPP-4, either in plasma and tissues, as also suggested by the fact that, in DPP-4-deficient rats, the amount of linagliptin in tissues increases linearly with dose and is eliminated rapidly whereas, in wild-type rats, increase in tissues is less dose proportional and its peripheral decline is much slower compared to DPP-4-deficient rats (Retlich et al., 2009). Following administration of a low-dose of linagliptin, AUC is significantly reduced and terminal half-life is significantly shorter in DPP-4 deficient rats compared to wild-type animals (Retlich et al., 2009). Hence, binding to DPP-4 in plasma and tissues potently affects linagliptin pharmacokinetics leading to a non-linear profile, a long terminal half-life, and a prolonged exposure to the drug. Based on these observations, and as also confirmed in human studies, tissue accumulation of linagliptin is unlikely and steady state conditions are reached very quickly.

Metabolism does not primarily affect linagliptin that is rather mainly eliminated unmodified. After repeated oral administration, the great part of linagliptin is excreted through the intestinal tract, a major part through the bile and a minor component (about 12%) directly into the gut, independently of biliary excretion (Fuchs et al., 2009b). In contrast only traces are found in urine (Fuchs et al., 2009a,b). Furthermore, following a single oral administration, the majority of drug is eliminated within 24 h, although about 4% is still present after 96 h.

### In vivo studies in animal models

Administration of linagliptin to Han Wistar rats produces inhibition of DPP-4 in plasma that appears after 30 min, is maintained 7 h after treatment and is still present at 24 h. Maximal effects are observed with doses of 3 and 10 mg/kg (90% inhibition), whereas more than 70% inhibition is achieved following 1 mg/kg. Calculated ED50 values are 0.3 and 0.9 mg/kg, 7 and 24 h post-dose, respectively (Thomas et al., 2008).

If inhibition of plasma DPP-4 activity is compared with that achieved using the same dosage of other DPP-4 inhibitors, linagliptin exhibits a similar efficacy vs. other compounds, at the 7 h time point. An exception is sitagliptin, that appears less effective at both time points examined, 7 and 24 h. In contrast, at 24 h, linagliptin exhibits a sustained and maximal inhibition of DPP-4 activity, not observed with the other drugs (Thomas et al., 2008).

Similar to other DPP-4 inhibitors, linagliptin (1 mg/kg) reduces blood glucose levels and suppresses glucose AUC between 0 and 120 min (about 50% reduction), when administered 45 min before glucose challenge. In contrast to other compounds, linagliptin maintains this effect (between 20 and 30% AUC reduction) when administration of the drug precedes by 16 h glucose load (Thomas et al., 2008).

In diabetic Zucker rats, administration of linagliptin 30 min or 24 h before an oral glucose tolerance test (OGTT) reduces blood glucose peak levels and plasma glucose AUC. This effect is accompanied by increase in GLP-1 and insulin levels (Thomas et al., 2008). In animals treated with linagliptin, while the increase of GLP-1 levels precedes glucose load and is maintained thereafter, insulin levels are not elevated in the fasting state, but sharply increase after feeding.

In an established animal model of diabetes, obtained with administration of a low-dose streptozotocin (STZ) and high-fat diet (HFD), the HFD/STZ rat exhibiting both hyperglycemia and hyperinsulinemia, oral daily administration of linagliptin (1, 3, 10 mg/kg), for 4 weeks, produces a dose-dependent inhibition of plasma DPP-4 (by 59, 78, and 87% with increasing doses) vs. levels at time 0, when measurements are carried out at the end of the study period (day 27), 21 h after last administration of the drug (Thomas et al., 2009). Reduction of DPP-4 activity is paralleled by decrease in blood glucose concentrations, glucose AUC, and post-prandial glucose levels, the latter indicating improved glucose tolerance (Thomas et al., 2009). An increase in active GLP-1 is also observed following multiple, once daily dosing of linagliptin, with no change of insulin levels. Furthermore, daily administration of linagliptin causes a significant, dose-dependent decrease of HbA1c with changes of 0.8, 0.9, and 1.0% at linagliptin doses of 1, 3, and 10 mg/kg, respectively.

Similar effects after chronic treatment with linagliptin are observed in a more drastic model of diabetes, the Zucker diabetic rat that exhibits marked insulin resistance, progressive beta-cell failure with age and a severe evolution of the disease. In this animal model, linagliptin acutely reduces glucose concentrations after OGTT and elevates GLP-1 levels, differently from vildagliptin, in a sustained and long-lasting manner (Thomas et al., 2008) without affecting basal insulin levels. In Zucker diabetic rat, chronic treatment with linagliptin does not cause change in body weight, but reduces cumulative food intake by 6%, in contrast to vildagliptin that does not modify this parameter.

In NOD mice that spontaneously develop a form of insulin-dependent diabetes caused by an autoimmune T cell-dependent destruction of islets, thus resembling human type 1 diabetes, linagliptin affects all the above described parameters. Specifically, exposure to linagliptin, given with food at doses of 3–10 mg/kg for 8 weeks, delays the onset and reduces the incidence of diabetes; it in fact induces reduction of basal blood glucose levels, decreases water intake and enhances GLP-1 levels. In addition, it significantly increases beta-cell mass in the pancreas without modifying the non-beta-cell component (Jelsing et al., 2012).

Interestingly, recent evidence suggests a positive interaction of linagliptin with an inhibitor of sodium-glucose co-transporter 2 (SGLT2) that is emerging as a novel target for treatment of T2DM. In genetically diabetic mice, linagliptin improves in fact the effects induced by the SGLT2 inhibitor, not only in terms of glycemic control and islet function, but also with regards to the expression of genes associated with the inflammatory response (Chen et al., 2012).

### Additional effects of linagliptin in animals models

The effects of linagliptin are not restricted to direct control of glycemic levels. As already mentioned, linagliptin in fact increases beta-cell mass component in the pancreas (Jelsing et al., 2012). In human cell islets *in vitro*, linagliptin (100 nM for 48 h) protects from apoptosis induced by exposure to insults such as palmitic acid, interleukin-1/interferon-γ, or hydrogen peroxide (Shah et al., 2010), restores beta-cell function and reverts oxidative stress responsible for islet dysfunction.

When administered, in a period comprised from 4 weeks to 3 months (3 or 30 mg/kg), to diet-induced obese rats and HFD-fed mice, linagliptin significantly reduces plasma leptin levels, as well as hepatic steatosis, measured as hepatic red oil staining and/or by magnetic resonance spectroscopy (Kern et al., 2012; Klein et al., 2012). The latter effect is particularly relevant as liver fat content correlates with metabolic disturbances, and signs of improved hepatic steatosis, as those observed following linagliptin, can be predictor of a better controlled glucose homeostasis. In addition, in these obese mice, high doses of linagliptin significantly reduce inflammatory response in adipose tissue, as measured by the number of infiltrating macrophages, and negatively affect the expression of several genes related with fatty acid synthesis and oxidation and primarily involved in inflammation (Kern et al., 2012). It is highly plausible that all these effects on lipid metabolism significantly contribute to the global improved insulin sensitivity induced by linagliptin. In obese rats, however, linagliptin does not modify total body weight, cumulative food intake or total body fat (Klein et al., 2012), but interestingly, it reduces weight regain following exenatide withdrawal (Vickers et al., 2012).

Linagliptin also seems to be endowed with vascular protective properties as it induces vasodilation in isolated aortic rings under basal conditions, in a concentration-dependent manner. This effect is mimicked by vildagliptin, but not by other DPP-4 inhibitors. It also affects vascular dysfunction-related reactive oxygen species and inflammation, thus exhibiting anti-oxidative and anti-inflammatory properties (Kröller-Schön et al., 2012).

Of note, linagliptin exhibits a protective effect against diabetes-dependent renal impairment. In diabetic endothelial nitric oxide synthase (eNOS) knockout mice, an established model of diabetic nephropathy, linagliptin, given as add-on therapy to the standard treatment telmisartan, improves albuminuria, a predictor of diabetic nephropathy, and reduces plasmatic levels of osteopontin, a marker of vascular calcification. These effects are accompanied by reduction of glomerulosclerosis and renal oxidative stress, as measured by accumulation of malondialdehyde, as well as reduction of tumor necrosis factor α (TNF-α) levels, a marker of systemic inflammation (Alter et al., 2012).

Inhibition of DPP-4 has been reported to have beneficial effects on protection from myocardial ischemic damage and ensuing improvement of cardiac function. Among possible mechanisms of action, a reduced degradation of myocardial SDF-1α, a substrate for DPP-4, with subsequent increased recruitment of circulating CXCR-4+ stem cells that may favor tissue repair. Alternatively, increased incretin acting at its receptor could activate pathways improving tissue repair. Treatment with linagliptin (given once daily for 7 days, starting 2 days before ischemia/reperfusion) induced less severe histological features of cardiac damage and reduced infarct size and myocardial fibrosis after ischemia reperfusion in rats (Hocher et al., 2012b). A similar reduction of infarct size is observed when linagliptin treatment is started 4 weeks before ischemia/reperfusion and is prolonged for 8 weeks thereafter. Linagliptin treatment also increased the expression of CD34, c-kit, CXCR-4, and SDF-1α, suggestive of enhanced recruitment of CXCR-4+ circulating progenitor cells that may be responsible for a better reparative process (Hocher et al., 2012b).

In *ob/ob* mice, that are known to exhibit delayed and impaired wound healing, treatment with linagliptin (3 mg/kg/day) while reducing hyperglycemia, improves wound closure and wound morphology. However, even at very high doses (30 mg/kg/day), it does not affect wound healing process in normoglycemic mice. The effects observed in *ob/ob* mice are accompanied by reduced influx and persistence of polymorphonuclear neutrophils, as well as infiltration of macrophages in wounds (Schürmann et al., 2012). Such effects appear extremely important and identify a potential additional effect of linagliptin. Reduction of hyperglycemia in fact does not necessarily correlate with improvement of wound healing. In addition, the high expression of DPP-4 in the skin and its down-regulation following injury suggest a role for the enzyme in reparative processes. Interestingly enough, in *ob/ob* mice, DPP-4 expression in the skin is absent at early times following injury, but is increased in the late phases of wound repair (Schürmann et al., 2012).

Finally, very recent data have shown that treatment with linagliptin produces a neuroprotective effect, independent of its glucose lowering action. Specifically, in a model of stroke induced by middle cerebral artery occlusion in normal and diabetic mice, linagliptin is effective in reducing stroke volume (with a greater effect in normal mice) and in increasing the number of viable neurons in the peri-infarct area (Darsalia et al., 2012).

## Studies in Humans

### Pharmacokinetics

First-human study was carried out to test pharmacodynamic and pharmacokinetic features as well as safety and tolerability of linagliptin (Hüttner et al., 2008). A single dose of oral linagliptin ranging from 2.5 to 600 mg/day produced different *t*_max_ values, from 0.7 to 3 h, depending on the dose. The 5 mg dose resulted in a *C*_max_ of 5.71 nmol/L, *t*_max_ of 1.47 h, *t*_1/2_ of 69.7 h, and very limited excretion in urine. In dose groups comprised between 5 and 50 mg, the terminal half-lives were approximately 70–80 h (69.7–79.9) whereas they ranged from 128 to 184 h in dose groups from 100 mg and above (Hüttner et al., 2008). Comparable pharmacokinetic profiles were observed in type 2 diabetic patients following single administration of linagliptin (2.5, 5, and 10 mg). Linagliptin was rapidly absorbed with a *t*_max_ of approximately 1.5 h. AUC and *C*_max_ increased with dose, but, as already observed in healthy subjects (Hüttner et al., 2008), the increase was less than dose proportional. This is likely due to high-affinity saturable binding of linagliptin to DPP-4. At low concentrations (1 nmol/L), in fact, binding of linagliptin to plasma protein is complete (99%) but it decreases to 70–80% at concentrations higher than 100 nmol/L (Fuchs et al., 2012). At low plasma concentrations, linagliptin free fraction is thus very low whereas, at higher plasma concentrations, linagliptin saturates DPP-4, its free fraction increases and pharmacokinetic parameters, including volume of distribution and clearance, are modified. As a consequence, terminal half-life is long, ranging from 110 to 130 h, depending on the dose administered. However, daily administration induced only moderate accumulation and a steady state level was yielded after 4–6 days following 1, 2.5, and 5 mg dose (Heise et al., 2009). Linagliptin showed a low rate of hepatic metabolism and renal excretion was minimal, being less than 1% on day 1 and between 3 and 6% on day 12 (Heise et al., 2009).

Food intake does not modify linagliptin pharmacokinetics in a clinically relevant manner. In fact, in subjects administered a high-fat meal, AUC_0–72_ of linagliptin was similar to that of control subjects. Although *t*_max_ was delayed by about 2 h (from 1.02 to 2.99 h) and *C*_max_ was reduced by 15%, these changes did not affect the overall exposure to the drug and did not modify its pharmacodynamics (Graefe-Mody et al., 2011c). It can then be concluded that linagliptin can be taken independent of meals as food does not seem to have any clinically relevant effect on the efficacy of the drug.

### Pharmacodynamics

Linagliptin induced marked inhibition of DPP-4 activity in both healthy subjects (Hüttner et al., 2008) and type 2 diabetic patients (Heise et al., 2009). According to Hüttner et al. (2008), a dose as low as 2.5 mg linagliptin caused about 70% inhibition of DPP-4 activity, whereas almost complete inhibition was observed at doses above 25 mg. The time needed to achieve maximum inhibition was variable depending on the dose and was about 3 h with the lowest doses. The inhibition was long-lasting and DPP-4 activity did not return to baseline levels after 96 h. In diabetic patients, with the 5 mg dose, 92.3% DPP-4 inhibition was observed at steady state and over 80% inhibition over a 24 h-interval after drug intake was still present (Heise et al., 2009). This level of inhibition (>80%) was maintained also after long-term treatments (12 and 24 weeks), when mean linagliptin trough concentrations were constant over time, at concentrations around 6.5 nmol/L (Del Prato et al., 2011). When patients were subjected to OGTT, linagliptin significantly reduced plasma glucose levels in a dose-dependent manner, starting at the 2.5 mg dose. This effect was present at 26 h, but was more pronounced at steady state, measured at 13 days (Heise et al., 2009).

### Pharmacokinetics and pharmacodynamics in patients with hepatic or renal impairment

In a phase I, parallel group-comparison study, 5 mg/day linagliptin was administered for 7 days to healthy controls and subjects with mild, moderate, and severe hepatic impairment. None of the groups showed significant changes in linagliptin pharmacokinetic parameters (AUC, *C*_max_, and *t*_max_), so that no dose adjustment is required in these patients. In fact, about 80% DPP-4 inhibition was present in all groups examined (Graefe-Mody et al., 2012). This observation appears particularly relevant in view of the potential correlation of type 2 diabetes with altered hepatic function and mainly with respect to the predominant non-renal excretion of linagliptin.

Pharmacokinetic profiles following linagliptin administration were investigated, both after single dose and at steady state, in patients with renal impairments of various degrees. Absorption of linagliptin after single and multiple dosages was similar to that observed in healthy and diabetic subjects, i.e., approximately or less than 3 h to reach maximal plasma concentrations. In addition, plasma concentrations 24 h after a single dose were similar in patients with mild, moderate, and severe renal impairment and were not modified by the concomitant presence of type 2 diabetes. AUC and maximal plasma concentrations at steady state were similar or only slightly increased in patients with moderate renal impairment when compared to healthy controls. In addition accumulation *t*_1/2_ of linagliptin following multiple 5 mg/day dose did not show any significant change in control and diabetic subjects, regardless of renal function (Graefe-Mody et al., 2011b).

Linagliptin present in urine was very low, around 1% in healthy controls, and reached only 4% in patients with renal impairment. Renal dysfunction did not modify linagliptin pharmacodynamics, as about 80% inhibition of DPP-4 was present, irrespective of renal function conditions.

These features make linagliptin unique among DPP-4 inhibitors as all other compounds are mainly excreted by the kidney and exhibit modified kinetics under conditions of renal impairment. After a single oral administration, approximately 87% of sitagliptin, 75% of saxagliptin, and 85% of vildagliptin are excreted in urine. The three drugs show profound changes in their plasma levels with increasing renal impairment, exhibiting already a twofold increase when a moderate renal dysfunction is present (Graefe-Mody et al., 2011b).

## Clinical Studies

A detailed report of all the clinical studies that have been carried out with linagliptin is beyond the scope of present review. Linagliptin has in fact shown a clinical efficacy that is comparable to that observed with other DPP-4 inhibitors. A summary of the clinical trials with linagliptin is reported in Table 1.

**Table 1:** **Summary of major results obtained with linagliptin in clinical studies**.

Treatment	Adjusted mean (SE) change from baseline in HbA1c (%)	Adjusted mean (SE) change from baseline in FPG (mmo/l)	Achievement of target HbA1c <7% (% of patients)	Reference
**4 WEEKS OF TREATMENT**				
Linagliptin, 2.5 mg (*n* = 26)	−0.31*	−16.6**	–	Forst et al. (2011)
Linagliptin, 5 mg (*n* = 16)	−0.37	−21.4	–	
Linagliptin 10 mg (*n* = 19)	−0.28	−16.6	–	
Placebo (*n* = 16)	–	+3.2	–	
**24 WEEKS OF TREATMENT**				
Linagliptin, 5 mg once daily (*n* = 336)	−0.44 (±0.05)	−0.5 (±0.1)	25.2	Del Prato et al. (2011)
Placebo (*n* = 167)	+0.25 (±0.07)	+0.8 (±0.2)	11.6	
**18 WEEKS OF TREATMENT**				
Linagliptin, 5 mg once daily (*n* = 151)	−0.39 (±0.14)	−0.7 (±0.3)	27.9	Barnett et al. (2012)
Placebo (*n* = 76)	+0.21 (±0.16)	+0.4 (±0.3)	15.1	
**26 WEEKS OF TREATMENT**				
Linagliptin, 5 mg once daily (*n* = 159)	−0.13 (±0.7)	−0.3 (±0.1)	30.2	Kawamori et al. (2012)
Linagliptin, 10 mg once daily (*n* = 160)	−0.19 (±0.7)	−0.4 (±0.1)	34.4	
Voglibose, 0.2 mg tid (*n* = 162)	+0.19 (±0.7)	+0.1 (±0.1)	22.2	
**12 WEEKS OF TREATMENT**				
Placebo (*n* = 80)	+0.63 (±0.08)	+0.4 (±01)	10.0	
**12 WEEKS OF TREATMENT**				
Linagliptin, 1 mg once daily (*n* = 65)	−0.15***	−0.36***	15	Forst et al. (2010)
Linagliptin, 5 mg once daily (*n* = 66)	−0.48	−1.22	15	
Linagliptin, 10 mg once daily (*n* = 66)	−0.42	−0.90	21	
Glimepiride, 1 mg once daily (*n* = 65)	−0.90	–	–	
Placebo (*n* = 71)	+0.25	+0.7	1.4	
(Add-on therapy to metformin)				
**24 WEEKS OF TREATMENT**				
Linagliptin, 5 mg once daily (*n* = 524)	−0.49 (±0.04)	−0.6 (±0.1)	26	Taskinen et al. (2011)
Placebo (*n* = 177)	+0.15 (±0.06)	+0.6 (±0.2)	9	
(Add-on therapy to metformin)				
**24 WEEKS OF TREATMENT**				
Linagliptin, 5 mg once daily (*n* = 142)	−0.5 (±0.1)	−0.5 (±0.2)	–	Haak et al. (2012)
Metformina, 500 mg bid (*n* = 144)	−0.6 (±0.1)	−0.9 (±0.2)	–	
Metformina, 1000 mg bid (*n* = 147)	−1.1 (±0.1)	−1.8 (±0.2)	–	
Linagliptin, 2.5 mg + metformina 500 mg bid (*n* = 143)	−1.2 (±0.1)	−1.8 (±0.2)	–	
Linagliptin, 2.5 mg + metformina 1000 mg bid (*n* = 143)	−1.6 (±0.1)	−2.7 (±0.2)	–	
Placebo (*n* = 72)	+0.1 (±0.1)	+0.6 (±0.3)	–	
**24 WEEKS OF TREATMENT**				
Linagliptin, 5 mg + pioglitazone 30 mg once daily (*n* = 130)	−1.06 (±0.06)	−1.8 (±0.1)	30.5	Gomis et al. (2011)
Placebo + pioglitazone, 30 mg once daily (*n* = 259)	−0.56 (±0.09)	−1.0 (±0.2)	42.9	
**24 WEEKS OF TREATMENT**				
Linagliptin, 5 mg once daily (*n* = 793)	−0.72 (±0.03)	−0.3 (±0.1)	29.2	Owens et al. (2011)
Placebo (*n* = 265)	−0.10 (±0.5)	+0.4 (±0.1)	8.1	
(Add-on therapy to metformin and a sulphonylurea)				

*SE, standard error; HbA1c, glycated hemoglobin; FPG, fasting plasma glucose*.

*Placebo-corrected mean change in HbA1c; **mg/dl; ***mean (+SE) change from baseline.

### Co-administration of linagliptin with other anti-diabetic drugs

No reciprocal interaction was evident when linagliptin and metformin were administered together (Graefe-Mody et al., 2009). Metformin *per se* did not modify DPP-4 activity and did not affect inhibition of the enzyme induced by linagliptin in healthy subjects. No clinically relevant pharmacokinetic interactions at steady state between the two drugs were observed. Metformin increased AUC by 20% and *t*_max_ of linagliptin by about 50%, but these effects are unlikely to have clinical relevance. Conversely, linagliptin co-administration only slightly reduced metformin *C*_max_ and delayed its *t*_max_.

A randomized open-label, two-way crossover study examined the effects of co-administration of multiple oral doses of linagliptin (5 mg/day × 6 days) and a single dose of glyburide (1.75 mg/day administered for 1 day) on the reciprocal pharmacokinetic parameters of the two drugs. Glyburide did not affect any pharmacokinetic feature of linagliptin and conversely, linagliptin slightly reduced glyburide AUC and *C*_max_ without achieving a clinically relevant effect (Graefe-Mody et al., 2011d). As glyburide is a recognized substrate of CYP2C9 and CYP3A4, this lack of interaction confirms that linagliptin does not modify the activity of these metabolic enzymes.

Linagliptin did not either affect pharmacokinetics of pioglitazone when the two drugs were administered together. This may appear particularly significant since pioglitazone is a substrate for CYP2C8. Hence, pioglitazone (45 mg/day) did not modify linagliptin (10 mg/day) AUC and *C*_max_, whereas linagliptin only slightly reduced pioglitazone *C*_max_ (Graefe-Mody et al., 2010b). The two drugs appear fully administrable in combination with no relevant pharmacokinetic interaction.

### Interaction of linagliptin with other drugs not directly correlated with diabetes

An open-label, multiple-dose study examined, in healthy subjects, the pharmacokinetic interaction of linagliptin (10 mg/day) and simvastatin (40 mg/day), that is classified as a sensitive substrate of CYP3A4. Changes in CYP3A4 activity may significantly modify simvastatin metabolism, leading even to the occurrence of severe adverse effects. Co-administration of linagliptin increased (by 10–30%) both exposure and *C*_max_ of simvastatin and its active analog simvastatin acid (Graefe-Mody et al., 2010a), without however achieving clinical relevance in terms not only of pharmacokinetic parameters, but also of safety and appearance of adverse effects during co-administration.

Eighteen healthy subjects were enrolled to evaluate the reciprocal pharmacokinetic interaction of linagliptin and warfarin, a CYP2C9 substrate. No pharmacokinetic parameter of linagliptin, S-warfarin, and R-warfarin was modified and co-administration did not significantly change the coagulation parameters prothrombin time (PT) and international normalized ratio (INR; Graefe-Mody et al., 2011a). Together with the study in which linagliptin was co-administered with glyburide (Graefe-Mody et al., 2011d), this study supports the concept that linagliptin does not significantly affect CYP2C9 activity and that substrates for this enzyme, such as warfarin, can be administered in conjunction with linagliptin without dose changing.

To test the interaction of linagliptin with drugs that act as substrate for P-glycoprotein, an open-label cross over study enrolling 20 healthy subjects was carried out to evaluate co-administration of linagliptin and digoxin (Friedrich et al., 2011b). *In vitro* studies have in fact indicated linagliptin as a weak *P*-glycoprotein inhibitor (Ishiguro et al., 2013). There was no significant change in digoxin pharmacokinetics and all parameters were within the bioequivalence range 80–125%, a critical aspect due to the low therapeutic index of digoxin.

Due to the wide use of anti-diabetic drugs in women of fertile age, the potential of impact of linagliptin on pharmacokinetics of an oral contraceptive combination composed of ethinyl estradiol (30 μg) and levonorgestrel (150 μg) was examined in an open-label, multiple-dose study carried out in healthy women. None of the parameters examined including both AUC and *C*_max_ at steady state were significantly affected by linagliptin co-administration, with values within the standard acceptance limit of bioequivalence 80–125% (Friedrich et al., 2011a).

## Safety and Tolerability

In a phase I study carried out by Hüttner et al. (2008), linagliptin was shown to be well tolerated in healthy male volunteers when administered in doses ranging from 2.5 to 600 mg per day. The overall incidence of adverse effect was similar in placebo- and linagliptin-treated subjects and there were no serious adverse effects (Hüttner et al., 2008). Hypoglycemia was never present and no changes of electrocardiographic or other cardiac function parameters could be detected (Hüttner et al., 2008). In this respect, a randomized, double-blind, placebo-controlled study was carried out in healthy subjects to evaluate the potential effect of linagliptin on QT interval. At the therapeutic dose (5 mg/day) and the 20-fold therapeutic dose (100 mg/day) administered, linagliptin did not cause any prolongation of the QT interval (Ring et al., 2011).

However, with regards to general cardiovascular risk in patients treated with linagliptin, a large meta-analysis evaluated cardiovascular events observed in eight phase III studies using linagliptin and different comparator anti-diabetic drugs (Johansen et al., 2012). It can be concluded that a reduction of cardiovascular events is observed in patients with type 2 diabetes treated with linagliptin vs. pooled comparators, suggesting a potential cardiovascular benefit of linagliptin.

Safety and tolerability data obtained with linagliptin in eight phase III clinical trials have been collected in a recent review article and data obtained from about 3,500 patients were analyzed altogether (Schernthaner et al., 2012). The overall incidence of adverse events and serious adverse events was similar in placebo- and linagliptin-treated patients (55 vs. 55.8 and 2.7 vs. 2.8%, respectively).

Hypoglycemia was the most important adverse effect occurring in 5.1% of patients receiving placebo vs. 8.2% of patients on linagliptin treatment. Severe hypoglycemia was absent in both groups. The higher incidence of hypoglycemia in linagliptin group was most likely attributable to the concomitant treatment with a sulfonylurea, as incidence of hypoglycemia was drastically reduced when the sulfonylurea was not allowed (0.6 vs. 1% with linagliptin and placebo, respectively). Among other adverse events, the gastrointestinal disorders were more common, occurring in 10.5% of linagliptin-treated patients, very similar to placebo–treated (11.4%). Diarrhea appeared in 2.1% of patients on either linagliptin or placebo.

Other adverse events occurring at a frequency >2% were infections of the upper respiratory tract, urinary tract or nasopharyngitis, headache, backpain, and hypertension. Minor adverse events, including increase of hepatic enzyme and serum creatinine, blood, and lymphatic disorders, occurred at an incidence <2% and were similar in patients treated with linagliptin or placebo. Cardiac disorders, mainly palpitation and tachycardia prevailed in linagliptin-treated patients, but always at an incidence <0.5% (Schernthaner et al., 2012).

An important issue that has been evaluated in several different studies is weight gain, as it represents a main adverse event with the use of other anti-diabetic drugs. DPP-4 inhibitors have generally a neutral effect on weight and, accordingly, the major part of studies on linagliptin reports no significant change of body weight or waist circumference at 12 or 24 weeks when the drug is administered alone (Hüttner et al., 2008; Del Prato, 2011; Del Prato et al., 2011), or in combination with other anti-diabetic drugs (Owens et al., 2011; Taskinen et al., 2011; Haak et al., 2012). Exceptions were provided by studies carried out by Forst et al. (2010, 2011) that reported a slight reduction in body weight after 4 or 12 weeks of treatment with linagliptin in monotherapy (Forst et al., 2011) or in addition to metformin (Forst et al., 2010). In contrast, an increase in body weight was reported after 24 weeks of treatment with linagliptin plus pioglitazone (2.3 kg) vs. pioglitazone plus placebo (1.2 kg; Gomis et al., 2011).

## Conclusion

Linagliptin is an oral, highly selective DPP-4 inhibitor causing over 80% DPP-4 inhibition throughout the 24-h dosing interval. It exhibits non-linear pharmacokinetic properties with a less than dose proportional profile and is almost completely eliminated via the enteric system, with less than 5% found in urine. This particular feature makes linagliptin unique among other DPP-4 inhibitors, as no dosage adjustment is required in patients with renal impairment and the drug can be used safely. The efficacy of linagliptin in improving glycemic control and reducing HbA1 has been analyzed in several different studies with linagliptin used in monotherapy or as add-on therapy to other commonly used anti-diabetic drugs. Finally linagliptin has a good tolerability profile, with the vast majority of adverse events of mild to moderate severity.

Besides the pharmacokinetic and clinical features of linagliptin, more impressively, preclinical studies have highlighted beneficial effects on the cardiovascular system, and in skin reparative processes that deserve further investigations.

## Conflict of Interest Statement

Maria Angela Sortino and Pier Luigi Canonico have been consultants of Boehringer Ingelheim Pharma GmbH. Tizian Sinagra has no conflict of interest to declare.
